# The Role of Ultrasound in Temporomandibular Joint Disorders: An Update and Future Perspectives

**DOI:** 10.3389/fmed.2022.926573

**Published:** 2022-06-20

**Authors:** Beatrice Maranini, Giovanni Ciancio, Stefano Mandrioli, Manlio Galiè, Marcello Govoni

**Affiliations:** ^1^Rheumatology Unit, Department of Medical Sciences, University of Ferrara, Ferrara, Italy; ^2^Department of Cranio-Maxillofacial Surgery, Unit of Cranio-Maxillofacial Surgery, University of Ferrara, Ferrara, Italy

**Keywords:** ultrasonography, temporomandibular joint, temporomandibular joint disorders, diagnostic imaging, articular disc, capsular width, joint pain

## Abstract

Temporomandibular joint (TMJ) disorder is the second most common chronic pain condition affecting the general population after back pain. It encompasses a complex set of conditions, manifesting with jaw pain and limitation in mouth opening, influencing chewing, eating, speaking, and facial expression. TMJ dysfunction could be related to mechanical abnormalities or underlying inflammatory arthropathies, such as rheumatoid arthritis (RA) or juvenile idiopathic arthritis (JIA). TMJ exhibits a complex anatomy, and thus a thorough investigation is required to detect the TMJ abnormalities. Importantly, TMJ involvement can be completely asymptomatic during the early stages of the disease, showing no clinically detectable signs, exposing patients to delayed diagnosis, and progressive irreversible condylar damage. For the prevention of JIA complications, early diagnosis is therefore essential. Currently, magnetic resonance imaging (MRI) is described in the literature as the gold standard method to evaluate TMJ. However, it is a high-cost procedure, not available in all centers, and requires a long time for image acquisition, which could represent a problem notably in the pediatric population. It also suffers restricted usage in patients with claustrophobia. Ultrasonography (US) has emerged in recent years as an alternative diagnostic method, as it is less expensive, not invasive, and does not demand special facilities. In this narrative review, we will investigate the power of US in TMJ disorders based on the most relevant literature data, from an early screening of TMJ changes to differential diagnosis and monitoring. We then propose a potential algorithm to optimize the management of TMJ pathology, questioning what would be the role of ultrasonographic study.

## Introduction

The temporomandibular joint (TMJ) is a bicondylar articulation of the ellipsoid variety (1). It is a synovial joint and thus it is susceptible to arthritis and related inflammatory conditions (2).

Following chronic low back pain, TMJ disorders (TMD) are the second most common musculoskeletal condition affecting approximately 5–12% of the population (3), causing chronic pain and even disability if untreated. Thus, a prompt diagnosis, before morphological degeneration occurs, is crucial (4, 5).

The Research Diagnostic Criteria for TMD classifies patients into three groups: (a) myogenous (sustained by muscular dysfunction, bruxism, abnormal posture, and myofascial conditions); (b) disk displacement or articular disk derangement; (c) articular causes (arthralgia, inflammatory arthritis, osteoarthritis, and less commonly ankylosis and neoplastic conditions) (6).

Although the most frequent causes of TMD are dental or orofacial phenomena, clinicians should not neglect inflammatory arthritis as a source of arthropathy (6), mainly rheumatoid arthritis (RA), psoriatic arthritis (PsA), and ankylosing spondylitis (AS) (7–9). Consequently, patients with known rheumatological conditions should be regularly screened for TMD, even if this assessment is not currently included in the routine screening and monitoring protocols (2).

Inflammation and increased vascularity are supposed to play a pivotal role in the pathogenesis of TMJ painful dysfunction (10). Moreover, TMJ represents a unique model to study bone changes in osteoarthritis (OA), because TMJ condylar articular surface is covered only by a thin layer of fibrocartilage, and the bone of the mandibular condyle is located just beneath the fibrocartilage, making it particularly vulnerable to inflammatory damage (11, 12).

The typical physical examination comprises evaluation for pain, stiffness, joint noises, and asymmetric or reduced mouth opening (13). Recently, published recommendations also encouraged detailed examination of masticatory muscles by palpation, as muscle tenderness may reveal an active disease (7, 14).

However, TMJ configures one of the most challenging joints to evaluate clinically, due to relatively uncommon evidence of swelling and paucisymptomatic conditions occurring during the early stage of the disease (15, 16). Thus, while certain abnormalities at the physical examination are strongly suggestive of TMJ involvement, their absence does not exclude it.

As there are no treatments to reverse the TMJ chronic damage once established, early diagnosis represents the only opportunity to prevent extensive and permanent joint derangements. Nonetheless, the current diagnosis, based on the Diagnostic Criteria for Temporomandibular Disorders (DC/TMD), confirms that TMJ degradation has already occurred, as documented in the standard imaging recommended protocols (computed tomography, CT, and magnetic resonance imaging, (MRI) (17). Therefore, the DC/TMD criteria are based on pre-existent condylar damage, namely surface erosions, osteophytes, or generalized sclerosis, mainly present in the later stages of the disease.

The purpose of this narrative review is to outline the role of ultrasonography (US) in the early diagnosis, differential diagnosis, patient reassessment, and monitoring of TMD. Furthermore, we want to explore the place of US in disease detection and follow-up appraisal, alongside the MRI and CT investigations.

To ensure a comprehensive update on the recent developments in this field, search strategies were adopted complying with recommendations for narrative reviews (18). We searched the PubMed and Embase databases up to March 2022.

Temporomandibular joint disorders, temporomandibular joint arthritis, temporomandibular joint dysfunction, temporomandibular joint osteoarthritis, temporomandibular joint disk, jaw disease, temporomandibular pain, temporomandibular joint ultrasound, ultrasound, sonography, and their respective MeSH terms were used as keywords. Specifically, we selected studies addressing the contribution of US in the diagnosis and prognostic outcomes, compared to the other imaging techniques, analyzing the advantage of US employment over MRI or CT. Only studies published in the English language were included, and the additional references quoted in these articles were also included when relevant.

## Methods

Our work is a narrative review. A comprehensive search of the literature published from inception to March 2022 was conducted. Two databases, PubMed and Embase, were utilized. Abstracts and titles were searched using keywords, MeSH terms as aforementioned, and subject headings, which were selected as they corresponded to the key characteristics of TMD, TMJ examination, and TMJ US that have been described in the introduction. The papers were then screened for eligibility: to be included, items needed to report studies that involved people with TMJ derangement or populations at risk for TMD. Papers that did not fit into the conceptual framework of this review or did not deal with the examination experience of TMD were excluded.

We grouped the studies according to the topic: imaging examination in TMD, US in TMD, TMD manifestations and differential diagnosis, and US in invasive procedures.

In addition, the references of relevant papers were hand-searched and their citations were examined. Only publications in English were considered.

**Figure 1:**
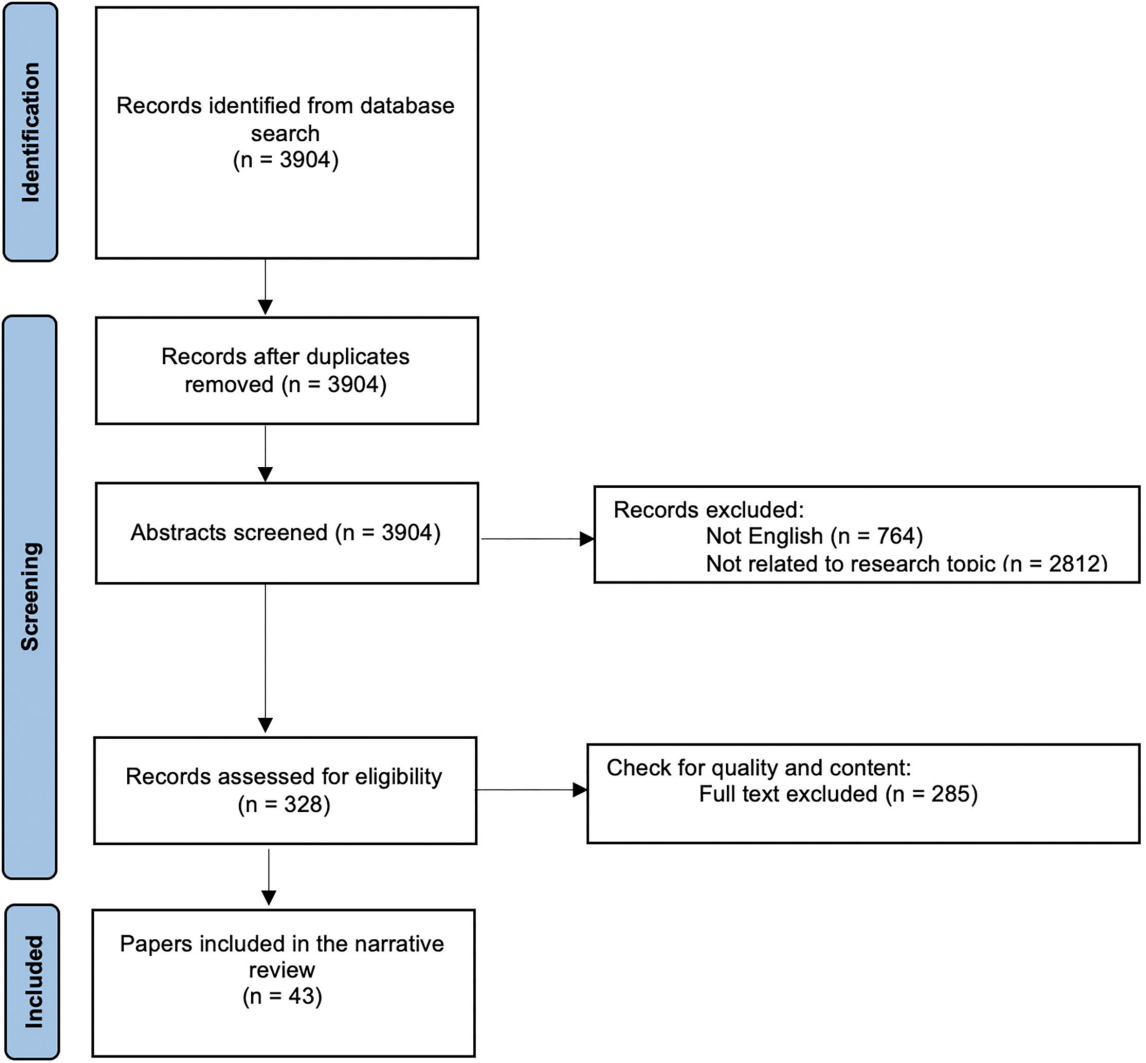
Flow diagram describing the inclusion decision of papers under the scope of this review.

Data from the selected papers were extracted. Figure 1 summarizes the selection and screening process: in total 43 articles were critically reviewed and consolidated for this literature review.

In Table 1, we summarize the key findings of the main articles employed for this narrative review.

**Table 1:** Key features of the studies.

**First author and reference in the manuscript**	**Year**	**Study population**	**Type of study**	**Topic**	**Main statement**
**IMAGING IN ASYMPTOMATIC / EARLY SYMPTOMS PATIENTS**					
Hayashi et al. (19)	2001	Elementary school children	Prospective	US vs. MRI and CT in early detecting TMJ involvement in JIA	Although US accuracy for the diagnosis of disk displacement is slightly inferior to that of MR or CT, authors assert US as a useful imaging method for longitudinal investigations of elementary school children.
Melchiorre et al. (20)	2010	JIA	Prospective	Clinical examination vs. US in early detecting TMJ involvement	Early stage oligoarticular JIA children are likely to have inflammation of the TMJs even in the absence of symptoms. US is a simple-to-use, noninvasive, radiation-free tool for the assessment and follow-up of TMD.
Muller et al. (21)	2009	JIA	Prospective	Clinical examination and US vs. MRI in early detecting TMJ involvement	None of the methods tested is able to reliably predict the presence or absence of MRI-proven inflammation of the TMJs.
Von Kalle et al. (22)	2015	JIA	Retrospective	CE-MRI in early detecting TMJ involvement	The degree of CE alone do not allow differentiation between TMJs with and without signs of inflammation. Thickening of the soft joint tissue seems to remain the earliest sign to reliably indicate TMJ arthritis.
Weiss et al. (23)	2008	JIA	Prospective	MRI vs. US in early detecting TMJ involvement	TMD are present in the majority of patients with new-onset JIA, even if normal jaw examination is present. MRI and US findings are not well correlated, and MRI is preferable for the detection of TMJ disease in new-onset JIA.
**IMAGING TECHNIQUES AND COMPARISON IN TMD**					
Ahmad et al. (24)	2009	TMD	Diagnostic criteria establishment	Development of image analysis criteria	Authors suggest assessing osteoarthritis using CT, and disc position and effusion using MRI. No mention on US.
Al-Saleh et al. (25)	2016	TMD	Systematic review	MRI vs. CT in detecting TMJ involvement	Very limited studies of MRI and CT to reach a conclusion. MRI better at disk position visualization.
Dong et al. (26)	2021	TMD	Prospective	Determining the optimal MRI sequences for TMD	The three optimal MRI sequences are oblique sagittal proton density-weighted imaging, oblique coronal T2-weighted imaging with closed mouth, and oblique sagittal T2-weighted imaging with opened mouth.
Friedman et al. (27)	2020	TMD	Prospective	US vs. MRI in detecting TMJ involvement	US is both a sensitive and a specific screening tool for TMD when used by an appropriately trained operator, with the exception of medially displaced discs. If TMJ assessment is found to be abnormal, the patient should be referred for MRI. If a component of medial disc displacement is suspected, MRI should be performed despite a normal screening US.
Hechler et al. (28)	2018	JIA	Systematic review	MRI vs. US in detecting TMJ involvement	Dynamic HR-US improves sensitivity and specificity compared to static, low-resolution US. Among TMJ changes (disk displacement, joint effusion, bony deformity), only joint effusion was appropriately assessed by multiple authors. US imaging following a baseline MRI can increase US sensitivity and specificity.
Kulkarni et al. (29)	2013	PsA	Case report	CT and X-ray in detecting TMJ involvement	CT and X-ray show erosion and resorption of the mandibular condyles, as well as calcification and osteophytic spurs in the joint space.
Landes et al. (30)	2007	TMD	Prospective	2D and 3D-US vs MRI in detecting TMJ involvement	3D-US in closed mouth position appears superior in diagnosing disk dislocation, and in overall joint degeneration. Sensitivity, accuracy and positive predictive value ameliorate if US is clinically applied prior to MRI.
Manfredini et al. (31)	2009	TMD	Systematic review	US vs. MRI, CT and clinical assessment in detecting TMJ involvement	US remains potentially useful as an alternative imaging technique for monitoring TMJ disorders, particularly the presence of intrarticular effusion (good accuracy). Better standardization of the technique is required, and normal parameters must be set.
Melchiorre et al. (32)	2003	RA, PsA	Prospective	MRI vs US in detecting TMJ involvement	US imaging can detect different pathological changes of TMJs and may be considered an important diagnostic tool.
Mupparapu et al. (33)	2019	RA	Systematic review	MRI vs. CT vs. PET in detecting TMJ involvement	PET used in conjunction with CT is the only imaging modality that can quantify TMJ inflammation in active RA disease.
Navallas et al. (34)	2017	JIA	ND	MRI in detecting TMJ involvement	MRI is the technique of choice for the study of TMJ arthritis. MRI is the only TMJ exam able to demonstrate bone marrow edema.
Sodhi et al. (35)	2015	RA	Case report	CT in early diagnosis	CT is a useful technique in diagnosing the bony changes (erosions) in the early phase of the disease.
Zwir et al. (36)	2020	JIA	Prospective	PDUS vs. MRI in detecting TMJ involvement	PDUS could be a useful screening exam to identify TMJ inflammatory activity. However, PDUS cannot replace MRI for the detection of TMJ inflammatory involvement.
**TMJ DISC DISPLACEMENT**					
Dong et al. (37)	2015	TMJ disc displacement	Meta-analysis	HR-US in detecting TMJ involvement	HR-US delivers acceptable performance when used to diagnose anterior disc displacement, being superior for the detection of anterior disc displacement without reduction rather than with reduction.
Emshoff et al. (38)	1997	TMD	Prospective	US in TMJ disc displacement	Both static and dynamic US modalities are insufficient in establishing a correct diagnosis of disk displacement.
Landes et al. (39)	2006	TMD	Prospective	3D-US vs. MRI in TMJ disc displacement	3D-US proves to be reliable for exclusion of disk degeneration compared with MRI, whereas the presence of such finding cannot be reliably diagnosed by 3D-US.
Li et al. (40)	2012	TMD	Systematic review and meta-analysis	US vs. MRI in TMJ disc displacement	The diagnostic efficacy of US is acceptable and can be used as a rapid preliminary diagnostic method to exclude some clinical suspicions. However, positive US findings should be confirmed by MRI. The ability of US to detect lateral and posterior displacements is still unclear.
Pupo et al. (41)	2016	TMJ disc displacement	Meta-analysis	Clinical examination vs. MRI in TMJ disc displacement	Clinical examination protocols have poor validity to diagnose disc displacement. MRI shows better results.
Severino et al. (42)	2021	TMD	ND	Clinical examination and MRI vs. US in TMJ disc displacement	US shows acceptable results in identifying bone structures. However, lower values of diagnostic efficacy were obtained for disc position during joint movements with respect to MRI images.
Tognini et al. (43)	2005	TMJ disc displacement	Prospective	US vs. MRI in TMJ disc displacement	US proves to be accurate in detecting normal disc position and the presence of abnormalities in disc-condyle relationship. US is not so useful for the distinction between disc displacement with and without reduction.
Westesson et al. (44)	1992	TMD	Prospective	Relationship between MRI effusion and clinical examination	TMJ effusion primarily occurs in joints with disk displacement and is strongly associated with joint pain.
**US IN TMD**					
Almeida et al. (45)	2019	TMD	Systematic review and meta-analysis	US in detecting TMJ involvement	US has acceptable capability to screen for disk displacement and joint effusion in TMD patients. For screening of condylar changes, ultrasound needs further studies using CT. More advanced imaging such as MRI can thereafter be used to confirm the diagnosis if deemed necessary.
Assaf et al. (46)	2013	JIA	Prospective	HR-US in detecting TMJ involvement	HR-US improves sensitivity and specificity in the detection of TMJ involvement, especially for the detection of condylar involvement in children with JIA (even if not all parts of the TMJ are visible on US).
Emshoff et al. (47)	2003	TMD	Prospective	HR-US in detecting TMJ involvement	US is an insufficient imaging technique for the detection of condylar erosion. Assessment of disc displacement without reduction may be reliably made with US.
Hu et al. (48)	2020	TMD	Systematic review and meta-analysis	US-guided arthrocentesis vs. conventional arthrocentesis in TMD	US-guided arthrocentesis may not improve postoperative pain and maximal mouth opening in the short term.
Jank et al. (49)	2007	JIA	Prospective	Clinical examination vs. US in detecting TMJ involvement	The significant correlation between pathologic US findings, duration of JIA, and the number of affected peripheral joints make US technique interesting for use as a diagnostic screening method.
Kim et al. (50)	2021	TMD	Prospective	US in detecting TMJ involvement	Capsular width is a risk factor for TMJ pain after adjusting for confounders. A refined and established protocol for standard examinations is needed.
Kundu et al. (51)	2013	TMD	Narrative review	US in detecting TMJ involvement	US is overall an acceptable diagnostic tool for detection of disc displacement (but MRI remains gold standard), condylar erosion and articular effusion.
Manfredini et al. (52)	2003	TMD	Prospective	US measures of TMJ capsular width (in mm) and MRI diagnosis of TMJ effusion	The critical US area is around 2 mm value for TMJ capsular width.
Parra et al. (53)	2010	JIA	Retrospective	US in joint injections	TMJ injections using sonographic guidance is a safe, effective and accurate procedure.
Rudisch et al. (54)	2006	TMD	Autopsy specimens	HR-US in detecting TMJ involvement	Condylar erosion is reliably assessed by HR-US, but the evaluation of disk position is less accurate.
Tonni et al. (55)	2021	JIA	Pilot study (TMD vs. healthy controls)	US in detecting TMJ involvement	Ultrasound can detect differences in the TMJ features between JIA patients and healthy patients. US might be used as a follow-up tool.
Varol et al. (56)	2008	TMD	Prospective	PDUS in TMJ internal derangements (vs. arthroscopic findings)	Arthroscopic synovitis with varying degrees of synovial vascularization was detected in all patients with positive PDUS exam.
**DIFFERENTIAL DIAGNOSIS IN TMD**					
Fan et al. (57)	2019	Pseudogout	Case report	Differential diagnosis of TMD, role of US-guided procedures	US-guided fine-needle aspiration is a reliable tool for diagnosing tophaceous pseudogout of TMJs.
Imanimoghaddam et al. (58)	2013	Myofascal pain	Case-control	Differential diagnosis of TMD	There is a significant difference between control and myofascial pain disorders groups in terms of visibility, widths, and echogenicity of masseter bands, which might be related to muscle inflammation.
Klasser et al. (59)	2009	Parotid gland tumor	Case report	Differential diagnosis of TMD	Parotid gland masses can be accompanied by pain and TMJ dysfunction, mimicking TMD, which may delay definitive diagnosis.
Matsumura et al. (60)	2012	Pseudogout	Case report	Differential diagnosis of TMD	Synovial chondromatosis with deposition of calcium pyrophosphate dihydrate may affect TMJs.
Poveda-Roda et al. (61)	2018	Myofascal pain	Case-control	Differential diagnosis of TMD	There is no statistically significant differences in masseter muscle width between chronic myofascial pain subjects and control subjects. The increase in width under maximum contraction is not significantly different between the groups.

*2D, two-dimensional; 3D, three-dimensional; CE, contrast enhancement; CT, computed tomography; HR-US, high-resolution ultrasound; JIA, juvenile idiopathic arthritis; MRI, magnetic resonance imaging; ND, not defined/not deductible; PDUS, power Ddoppler ultrasound; PET, positron emission tomography; PsA, psoriatic arthritis; RA, rheumatoid arthritis; TMD, temporomandibular disorders; TMJ, temporomandibular joint; US, ultrasonography/ultrasound*.

### Imaging Examination Techniques in TMJ Disorders: What Is the Current State of the Art in Ultrasound?

Although a large proportion of patients affected by TMJ arthritis are completely asymptomatic during the early stages of the disease (complaining of neither pain nor impaired TMJ function) and present a normal TMJ clinical examination (23), radiographic signs of TMJ damage may still be revealed even in the early phases of the disease.

Therefore, imaging acquires a pivotal role in the early assessment of TMJ changes, trying to prevent further impairment of TMJ. Additionally, a frequent instrumental follow-up is essential to evaluate the progression of the disease and response to the therapeutic approaches.

Conventional X-ray and CT scans reveal only advanced damage of TMJ arthropathy, but do not properly analyze the soft tissues, articular disk changes, and early or active signs of arthritis. Furthermore, even if CT provides accurate anatomic detail and it is thus beneficial in identifying surgical candidates (62), it lacks the dynamic imaging potential, and it employs a high radiation dose.

Therefore, MRI is now regarded as the current imaging “gold standard” for the evaluation of inflammatory processes in TMJ pathology, as it can identify both active arthritis changes as well as arthritic sequelae, showing a moderate-to-good reliability (21, 25, 26, 28, 41, 63–65).

MRI technique can detect acute signs of TMJs involvement, such as the presence of synovitis, which is better demonstrated by contrast-enhanced (CE) MRI sequences, joint effusion, and bone marrow edema. In addition, it reveals chronic signs of TMJs involvement, such as condylar changes, erosion, and abnormalities pertaining to the disk (28).

Despite many advantages, MRI also suffers some drawbacks. Namely, the time for image acquisition ranges from 20 to 45 min on average, and the exam requires an open-mouth position, which is particularly troublesome in patients experiencing TMJ pain. Besides, MRI allows mostly static image study, it necessitates the patient's collaboration, which may be difficult in the pediatric population or claustrophobic patients, and it is a high-cost procedure, not available in all centers. Moreover, MRI is contraindicated for patients with pacemakers, implantable cardiac defibrillators, and in the case of metallic foreign bodies (66).

Additionally, positron emission tomography (PET) and PET/CT represent novel technologies, which have shown good promises for the diagnosis and follow-up evaluation of TMD (33, 67). In fact, the maximum standardized uptake value (SUVmax) tends to be higher in the TMJ symptomatic patient or in the disease aggravation and decreases when TMD improves. In this regard, SUVmax may play a significant role, not only in detecting TMD, but also in evaluating the treatment response and measuring the TMD activity (68). However, the inflammatory activity in small joints such as TMJs has not been studied as extensively as in the large joints in PET studies (69), thus a careful interpretation is required.

To overcome these limitations, a promising alternative diagnostic tool seems to be represented by US, which is relatively inexpensive and potentially accessible in most outpatient clinics, after an adequate operator's training (51). The examination only takes 10–15 min ordinarily, a tolerable time even for the youngest patients; in the absence of radiation or any other risk, it is pain-free, and it allows dynamic real-time assessment, while the mouth is closed or opened, with the option of direct communication with the patient that can guide examination to painful regions. Furthermore, it does not require any sedation in children. However, it is unclear whether it can identify the active inflammation and arthritic sequelae as accurately as CE-MRI.

Consequently, there has been an intense effort to identify the sensibility and specificity of US as compared to MRI, particularly in the pediatric population, as several recent studies have shown that non-arthritic children can still present subtle findings on MRI consistent with TMJ arthritis, such as joint effusion and contrast enhancement, which may be possibly more easily and rapidly detected by US (16).

#### Ultrasound Protocol

**Figure 2:**
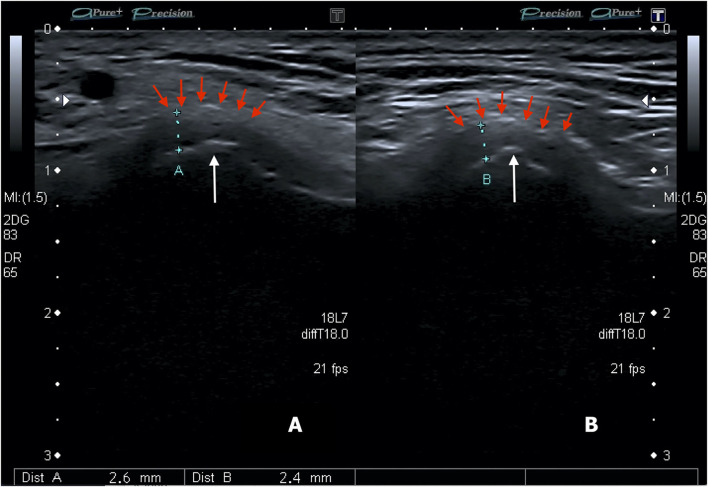
Transverse image of the right **(A)** and left **(B)** TMJs showing the condyle and capsular width (distance between markers). White arrows show the condylar process and red arrows show the articular capsule (Personal archive).

**Figure 3:**
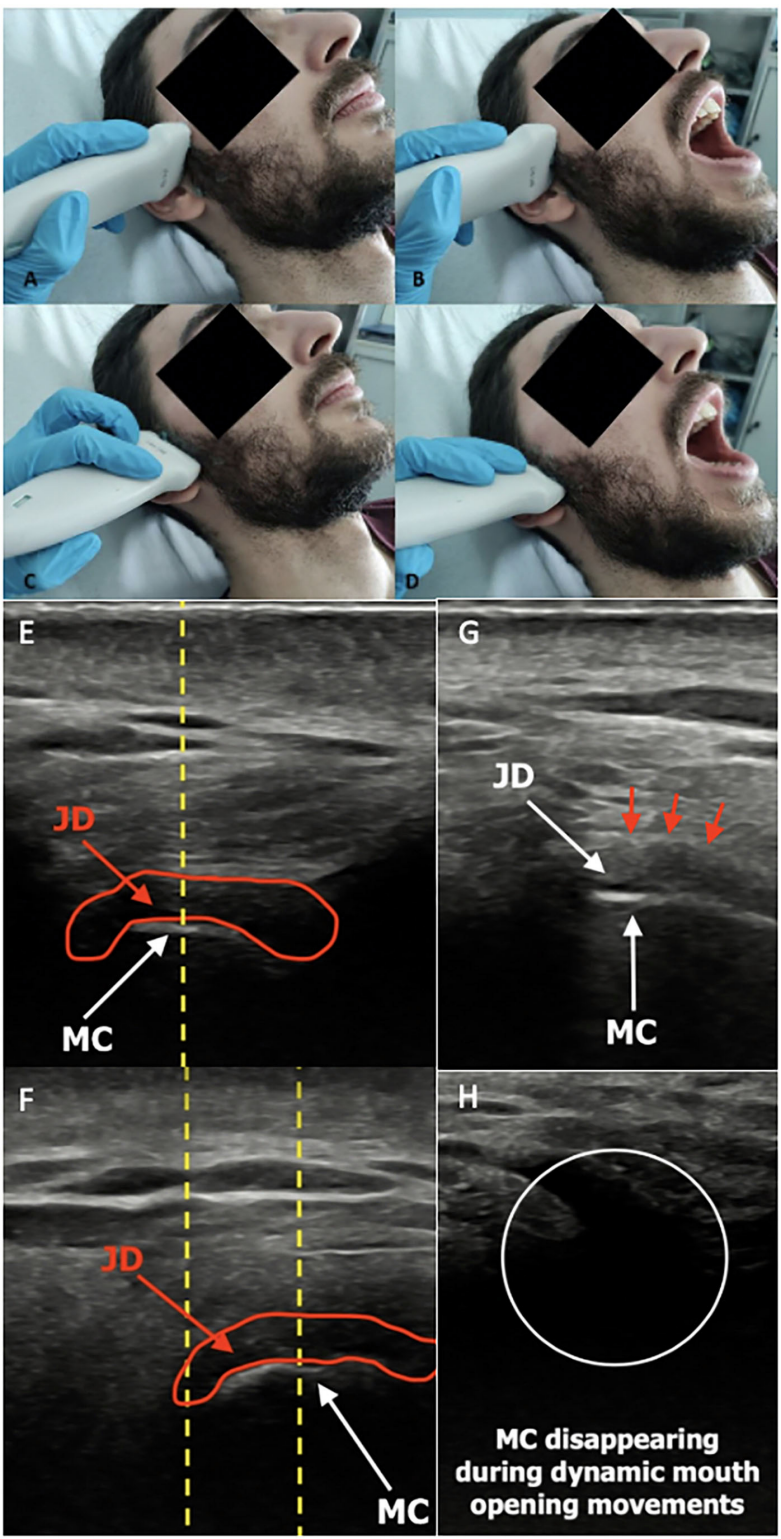
Conventional US transducer positions are parallel to the Frankfort horizontal plane (a plane connecting the highest point of the opening of the external auditory canal with the lowest point on the lower margin of the orbit) in closed-mouth **(A)** position and open-mouth **(B)** position, as well as parallel to the ramus of mandible, both in closed-mouth **(C)** and open-mouth **(D)** positions. Normal ultrasound image of TMJ in transverse sections in closed- **(E)** and open-mouth positions **(F)**. The normal ultrasound appearance of the articular disk in the sagittal plane is an inverted hypoechoic C-shaped structure, outlined by the red circle. During the mouth opening, the mandibular condyle translates anteriorly as defined by the distance between the center of the condylar oval at the two positions (yellow dotted line). Notably, the disk maintains a constant central appearance with respect to the center of the mandibular condyle in normal anatomy, while it may be displaced anteriorly or posteriorly in the pathological findings. Normal ultrasound image of TMJ in longitudinal closed-mouth **(G)** and open-mouth position **(H)**. Red arrows show the articular capsule. JD, joint disk; MC, mandibular condyle (Personal archive).

A common feature of TMJ involvement is synovitis, defined as a thickened synovia, joint effusion, and with or without an active synovial inflammation (55) (Figure 2). Afterward, arthritic changes may occur as reparative or destructive signs, cystic lesions, erosions, flattening of the articular condyle, as well as destructive changes of the articular disk and synovial structures (32, 46, 47, 49, 54). Currently, no different TMJ US findings characterizing condylar inflammation or damage have been described, as they may coexist. Moreover, to date, no defined TMJ US pattern has been reported to be peculiar to different TMD. The traditional US imaging protocol includes axial and coronal scans at closed- and open-mouth (55) (Figure 3).

The surface of the condyle is hyperechoic (high reflection of sound waves) and it appears white in the US images. The connective tissues, represented by a joint capsule, retrodiscal tissue, and muscles (lateral pterygoid and masseter), are isoechoic (intermediate reflection of sound waves) and appear heterogeneously gray in the US images.

However, the margin of the joint capsule highly reflects the US waves, generating a hyperechoic white line. These anatomic cavities are virtual because the opposite surfaces are in contact and usually not detectable unless effusion occurs (51).

Thus, the width of the synovial joint space is particularly relevant, because it may indirectly indicate the presence of a joint effusion, which is usually regarded as a sign of synovitis (46, 70).

The joint space width is measured from the cortical contour of the condyle to the articular capsule at different levels over the condylar cortical line (anterior and lateral levels). The coronal scan position is the most suitable to assess this measurement (46, 55, 70).

The US diagnosis of effusion has been favorably compared to the gold standard MRI technique, especially when the capsular width is above 1.950 mm in the adult population (52). In fact, current studies identify a critical TMJ capsular width of around 2 mm (31) and therefore focus attention on interobserver reliability. Moreover, the capsular width has been documented to be a risk factor for TMJ pain when adjusted for other confounders, thus it is an estimation with consequent clinical correlation (50).

In a pediatric study, Muller et al. (21) employed the same capsular width cut-off for the assessment of TMJ effusion, as had been applied for adults (2 mm), and this could explain a weak correlation observed between US and MRI. Thus, for the pediatric population, a cut-off level of 1.2 mm has been proposed (70), with better results in terms of the correlation between the US-assessed capsular width and MRI-assessed synovitis. In fact, a correct cut-off level is essential to avoid wrong discredit of US as an instrumental exam tool in TMD.

Conversely, only a few efforts have been made on MRI images to differentiate between the normal and abnormal TMJ effusions, defined as an area of high-signal intensity within the joint space on T2-weighted images (24). Only two studies attempted to address this question, defining the abnormal synovial joint space to be more than a line of high T2-signal along the joint surface (44, 71). A more recent study calculated a ratio of pixel intensity between TMJ synovia and the longus capitis muscle, suggesting this measure to be a reliable way to quantify synovial enhancement (72).

Importantly, the identification of synovial thickening alone in TMJ US might not indicate an ongoing active inflammation, but might rather represent a quiescent chronic disease. Considerably, the power Doppler (PD) images enable the diagnosis of an active TMJ inflammation through the detection of increased synovial vascularization that, while theoretically possible if contrast is used, is unlikely in MRI when performed with the standard practices (73). Additionally, the gadolinium-based contrast medium is generally considered safe, but it may be associated with adverse reactions, such as the idiosyncratic allergy-like reactions (74).

Few studies concluded that power Doppler US is a good technique for the assessment of synovial changes by microvascularization. A study by Varol and colleagues (56) assessed and confirms TMJ synovial vascularization both on US and arthroscopy.

Conversely, other studies showed no considerable differences between synovial inflammation obtained using power Doppler US or determined through MRI images, as the sensitivity is very low even in cases of the obvious inflammatory process, mainly because the deepest part of the TMJ cannot be assessed with this technique (34, 36). Nonetheless, the issue is not fully elucidated, as a lack of synovial enhancement on MRI may not exclude the joint inflammation as well (22).

Awareness should be raised regarding the increased signals of vascularity in pathological conditions aside from TMD. For instance, the majority of cases of pleomorphic adenoma present with color peripheral Doppler signal (75), and because TMJs are adjacent to the parotid gland, this element acquires particular relevance. At the same time, post-radiotherapy nasopharyngeal carcinomas patients showed a reduction in the TMJ disk thickness, an increase in condyle irregularity, and joint vascularity (76).

The main disadvantages of the US technique remain the long-learning curve and the fact that the examination is operator-dependent. Furthermore, ultrasound images present questionable anatomical validity, mainly because of the bone blockade barrier and the consequent inability of the US beam to identify all the local structures. Additionally, currently, only a few studies have been published upon this argument, limiting the evidence of data discussion. More recent works provide strong support for the use of conventional US techniques, and hopefully, future research will contribute to better knowledge on this topic, possibly reaching a definite consensus.

#### Ultrasound Sensitivity and Specificity

Far from being clarified, the sensitivity and specificity of US in recognizing TMJ changes are still debatable, due to the performance of US as compared to MRI (28, 51).

The main reason for this ongoing discussion is the wide heterogeneity of the study designs, in terms of the population [juvenile idiopathic arthritis (JIA), adult rheumatic conditions, non-rheumatic patients, and other TMJ derangements], US protocols, and considered parameters of specific acute and chronic TMJ changes (recognized as disk displacement, joint effusion, condylar deformity, even if only joint effusion has been appropriately investigated by multiple authors) (28). In addition to the above-mentioned aspects, there is a relative paucity of studies about the topic, which makes the comparison difficult and subject to possible biases.

Emshoff et al. (38) employed a transducer of 7.5 MHz, with a revealed sensitivity of 40–50%, and specificity of 70%. Sensitivity was found to decrease from closed- to open-mouth position, conversely, specificity increased from closed- to open-mouth position, but in both positions, the diagnostic accuracy was found acceptable.

Such findings may be explained by the medial disk displacement occurring after mouth opening, as the mandibular condyle and the glenoid cavity do not allow proper US propagation without appropriate adjustment of the probe in different planes, thus impairing the visualization of the articular disk. Nonetheless, this consideration does not apply to an ultimate 3D US, where the TMJ can be evaluated in different planes within the scan volume. The 3D US has also been found to have acceptable sensitivity and accuracy (39), but according to recent findings, it does not seem to significantly increase the reliability of the examination (51).

Similar results were found in other studies (77), and in particular, following progressive employment of transducer with higher frequency, of 10 MHz or more, allow a better sensitivity, ranging from 60% to 90% (30, 40, 42, 45). Specifically, a recent review (28) found that high-resolution US (HR-US), defined as a US resolution of 12 MHz or more, improves sensitivity and specificity in the detection of TMJ involvement as compared to low-resolution US (LR-US), defined as a US resolution of <12 MHz (28, 31). Moreover, a study by Jank et al. (49) found that HR-US is able to detect TMJ pathology even before clinical symptoms appeared, which is particularly relevant in the younger population to avoid damage accumulation. Melchiorre et al. (32) have found US quite useful even for the diagnosis of TMD in adult RA patients.

Few studies also illustrated the benefit of executing a baseline MRI to increase US accuracy, which can be reassessed during the follow-up, as attested by a reported increased US sensitivity and specificity parameters (28).

To the best of our knowledge, only one study compared the power of clinical examinations, MRI, and US imaging in TMD (21). The population study included JIA patients, and US was found to be the most specific of all tested methods, but the least sensitive, detecting only the most severely affected joints.

The studies comparing US with MRI in TMJ arthritis have determined a poor correlation between these modalities, with US potentially missing from 67% up to 75% of TMJ MRI-detected inflammatory changes (23, 78). Alongside, MRI contrast enhancement improves the detection of MRI TMJ inflammation from 35.7% to 86.7% (79).

A study by Weiss et al. (23), carried out on a population of children affected by JIA, compared MRI and US in detecting both the acute and chronic TMJ arthritic signs. For the acute inflammatory TMJ changes, the agreement between these two techniques was only 23%, and for chronic TMJ changes, the agreement reached 50%. These results indicate that MRI and US findings are not well correlated and that MRI shows a greater sensitivity for the detection of TMD.

Because of all the above-illustrated reasons, US is currently neither recommended as a screening method for early TMJ involvement nor for the monitoring purpose in the recent European League Against Rheumatism (EULAR) guidelines for JIA management (80), which claims MRI for both diagnosis and follow-up schedule.

However, the latter aspect has been debated in a recent review of the literature; although the paper concludes that US has low sensitivity in detecting joint effusion, its employment during follow-up monitoring is advocated by authors (28), highlighting again how current data do not answer the question whether US can assist MRI.

### TMJ Ultrasonography: Who, When, and What

#### Asymptomatic Patients

As previously mentioned, many patients affected by TMD can be totally asymptomatic during the early stages of the disease.

This is particularly remarkable in JIA, which is the most common childhood chronic rheumatic disease, encompassing different joint arthritis subsets, with an onset before the age of 16 years (81). TMJ dysfunction has been frequently reported in association with JIA (82, 83), with a prevalence of 17–87% according to different studies (84–87). Undiagnosed and consequently untreated disease can result in a variety of serious sequelae, particularly relevant for a population of growing children, including impaired facial development, dysmorphic facial features, mandibular asymmetry, micrognathia and retrognathia, and, in most severe cases, even condylar resorption, eventually require a total joint replacement. Melchiorre et al. (20) found that in the newly diagnosed JIA patients with US evidence of joint effusion, more than 95% did not complain of any joint pain. Remarkably, many of these patients were under the influence of anti-rheumatic drugs, which may hide the TMJ symptoms.

Even other inflammatory chronic arthropathies may present rather asymptomatic during the early stages of the disease.

RA is a chronic inflammatory joint disease, affecting mostly women. Clinical manifestations encompass symmetrical joint polyarthritis, possibly leading to progressive joint damage and irreversible disability (88). Thus, early diagnosis is deemed essential against the most undesirable outcomes. Albeit RA mainly affects the joints of the hands, wrists, elbows, knees, ankles, and feet, TMJ may be involved as well, even if less frequently. The literature data report from 4% up to 80% of RA patients clinically exhibit TMJ involvement (35). Morning stiffness may be present even at the TMJ site, along with decreased masticatory force (15). Morphologic alterations may be documented on conventional radiographs of the TMJ, ranging from 19% to 86% of RA patients (89). Occasionally, TMD may be the presenting manifestation of RA (90). Nevertheless, there are only few studies concerning TMJ and masticatory muscles involvement in patients with early RA; therefore, the relationship between TMD and the rheumatological condition remains unclear (15).

Interestingly, Crincoli et al. (15) carried out an early RA cohort (defined as patients who received RA diagnosis within 12 months); despite TMJ involvement, the study group complained less frequently about the TDM symptoms as compared to the healthy controls. Similarly, TMJ noises and opening derangement were significantly lower in the study group compared to the controls. These phenomena are probably explained by drug therapy, corticosteroids, or conventional/biological disease-modifying anti-rheumatic drugs (DMARDs), promoting downregulation of pro-inflammatory chemokines, and therefore masking the clinical picture.

Moreover, a study by Kroese et al. (91) demonstrated an increased risk of TMD in individuals with early RA, defined as a time limit of <3 years from symptoms onset (92), and at-risk of RA, as defined by the EULAR guidelines (93) (including joint symptoms <1 year, mainly located at metacarpophalangeal joints, with early morning stiffness and difficulties in making a fist, showing a positive squeeze test at joint examination), who should benefit from further TMJ examination and management.

Additionally, patients with PsA and, to a lesser extent, those with psoriasis (PsO) are equally more frequently affected by TMD as compared to the healthy subjects, and again, TMD may be the presenting manifestation of the rheumatic condition (8, 29, 94). Dervis et al. (95) showed TMJ dysfunction in 29% and 35% of patients with PsO and PsA, respectively.

TMJ involvement is also possible in AS, and it occurs in 22% of patients, but frequently most patients complain of no symptoms, so this is likely to be an underestimation (96).

Today, to the best of our knowledge, there are no conclusive data on TMJ involvement in the asymptomatic patients, nor in pediatric or in adult population affected by rheumatological conditions. Currently, TMJs are not included in routine rheumatological ultrasound screening protocols. The clinimetric questionnaires present no specific questions for TMJs, and patients very often underestimate the early symptoms in terms of pain and joint clicks and do not tell physicians about them.

Therefore, TMJ involvement may undergo underreporting. This is a huge gap that hopefully will be conceivably investigated in future research.

As early functional disorders of TMJ are often preclinical, in all the above-mentioned populations of patients, and particularly in children, US would be beneficial as an entry-level diagnostic screening tool, which is rapidly accessible and of relatively low cost. Patients found with suspicious TMJ alterations would then be directed to complete second-level investigations, such as MRI and CT. Due to the low sensitivity of US method, some patients will be considered devoid of TMJ changes at first US screening procedure, but would anyway be undiagnosed due to the current absence of guidelines suggesting MRI or CT early screening in these populations, while, for example, ultrasound and X-ray imaging is now considered the gold standard both for the early diagnosis and progression monitoring in many forms of osteoarthritis regarding other anatomical joints (97–99).

Remarkably, currently, as for US, no differences in MRI findings have been documented in JIA, RA, PsA, or PsO patients in the literature.

#### TMJ Ultrasound: Is a “Point-of-Care Ultrasonography” Possible?

As already explained, TMD is a frequent cause of orofacial pain, derived from trauma, rheumatoid disorders, and dental- and non-dental-origin causes. The reported TMJ pain can be regionally localized or generalized as myofascial pain (100), and sometimes other clinical conditions may mimic TMD.

As clinicians, we search for a quick precise diagnosis; therefore, we collect a careful anamnesis of pain characteristics and a complete clinical examination, but sometimes, we are still doubtful about the diagnosis. In some cases, US would come to the rescue, adding precious clues to address the diagnosis.

For example, heterotopic ossification has been reported to be associated with crystalline arthropathies and secondary systemic illnesses such as gout and chondrocalcinosis (101, 102). Deposition of calcium pyrophosphate dihydrate crystals occurs within and around TMJ, especially involving the articular cartilage and fibrocartilage, appearing as spotted hyperechoic signals on US images. Sometimes a marked destruction of the condyle with erosive changes may be observed in association (60).

Occasionally, especially in long-term gout disease, a TMJ palpable mass may be appreciated, and US may evidence a TMJ adjacent hypoechoic mass, corresponding to the tophaceous material (57, 103). In these cases, US can guide fine-needle aspiration for the histological confirmation of diagnosis (57).

The US-documented involvement of other joints with chondrocalcinosis is a clue to the diagnosis, while differential diagnosis includes synovial chondromatosis, synovial osteochondroma, and osteosarcoma (104).

Even in JIA children, it has been reported few cases with new bone formation rather than proper crystal deposition, and the new bone formation is frequently heterotopic, rather than condylar. In addition, in these cases, the heterotopic ossification appears to be intra-articular, rather than in the periarticular soft tissues (83).

TMJ referred pain may also be caused by salivary glands pathology, which is particularly relevant in the rheumatological population, as connective tissue diseases (CTD) may be associated with the enlargement of these glands. Indeed, salivary glands US is now advocated as a meaningful tool to be incorporated into the clinical evaluation among these patients, therefore many clinics are still performing it on patients with CTD (105).

A parotid gland swelling located in the deep lobe is a possible cause of TMD symptoms (59). This is due to the common vegetative innervation of the salivary glands and components of the TMJ. US is a dynamic exam, scanning different planes, therefore it may reveal a proximal enlarged parotid gland, or TMJ adjacent mass within the parotid gland presenting as a hypoechoic or heterogeneous US pattern, enabling further investigations to exclude possible tumor masses, such as pleomorphic adenoma of the parotid gland (106, 107). Therefore, even if the physician is not particularly skilled at salivary gland US, he or she can anyhow quickly identify a suspicious lesion, as it presents with a different echogenicity compared to the surrounding tissue, addressing the patient to further analysis.

TMJ tumors and pseudotumors are relatively infrequent, but usually present as orofacial pain, with a similar presentation to TMJ internal derangement. According to the literature, in adults, benign tumors primarily include chondroblastomas, osteoblastomas, osteochondromas, and osteomas, while metastatic tumors and sarcomas are the main malignant tumors (108). US may reveal a solid lesion, destroying the TMJ profile (109). Again, US may be considered a beginning examination, not necessarily requiring experienced ultrasonographers for justifying further investigations.

Rarely, temporal arteritis headache may mimic TMJ irradiated pain. In this case, only an expert rheumatologist in temporal arteries US is qualified to discern a halo sign, as a hallmark of giant cell arteritis, from TMJ derangement (110).

Focal myalgia caused by TMJ parafunction or myofascial pain may be another cause of regional pain (100). In recent years, studies using MRI and US in patients with masticatory muscle myalgia have frequently been reported (111). Few studies showed no statistically significant differences in the masseter muscle width between myofascial patients and control subjects (61), while others showed obvious US changes in the masseter muscle, especially in female myofascial syndrome patients (58). Muscle visualization technique is not currently performed in TMJ US, but would help in differential diagnosis, mainly in cases when maxillofacial surgeons find no conclusive elements at clinical evaluation, MRI or CT exams in patients reporting TMJ disturbs.

#### TMJ Disk Displacement

Features of TMD could derive from the articular disk changes.

In addition to the prior discussed disadvantages of US, another relevant one is the limited access to deep structures and in particular to the articular disk, derived by sound waves absorption by the head of the condyle and the zygomatic process of the temporal bone (31). Moreover, because the internal echoes of the articular disk are similar to those of the articular capsule, it is difficult to discriminate the articular disk from the articular capsule in the US images (19).

The evaluation of condylar and disk irregularities is a standard procedure in any assessment of MRI scans or conventional radiographs (19, 112). Some authors suggest that, with a few shrewdness by appropriately constantly adjusting the position of the transducer over examined structures, evaluation of the articular disk can be captured; however, US alone is likely to underestimate disk changes (46).

The disk is visualized only through a small space between the zygomatic process of the temporal bone and the head of the condyle. It is challenging to obtain satisfactory images, especially when the condyle rotates and translates from the closed-mouth position to the open-mouth position (51).

With the adjustment of probe position, disk thickness and shape can be assessed with US, and derangement may present as a hypoechoic inhomogeneity in the range of the articular disc. However, perforations and adhesions are not adequately visualized by US, nor is the medially displaced disk (27). Then, if a component of medial disk displacement is suspected, MRI should be performed directly, despite a normal screening US (27).

To the best of our knowledge, only one study in literature summarized the US power in the evaluation of condylar disk displacement: the overall sensitivity of HR-US compared to MRI across studies ranged from 0% to 100% and the specificity ranged from 63% to 100% (43). Sensitivity was found to be directly proportional to the resolution of the probe, as it increases following the increase of US resolution (37, 40, 47).

#### Invasive Procedures

Although US TMJ is mainly employed for the diagnosis of degenerative changes and synovitis, in recent years, US is growing as a supporting technique in therapeutic procedures, such as arthrocentesis procedures (sodium hyaluronate or steroid injections) to detect the disk and bone structures (113).

A study by Parra et al. (53) compared CT vs. US-guided TMJ injections. Needle placement was shown to be acceptable in 91% of US-guided procedures (75% required no needle adjustment, 16% only minor adjustment) and unacceptable in 9%, which means the needle required major readjustment.

A similar study using post-injection MRI to assess needle placement accuracy described a technical success of 100% (114).

Certainly, the US-guided procedures do not contain as much detail as the other advanced imaging techniques. A recent meta-analysis, in fact, showed that US may not improve postoperative pain and maximal mouth opening in short term after TMJ arthrocentesis, presenting scarce and conflicting results for any definite conclusion (48). On the contrary, US has no harmful effects and could be employed even in children and pregnant women, and this aspect may be considered in everyday practice.

### Monitoring TMJ Disease

A survey of the American Association of Oral and Maxillofacial Surgeons in managing and monitoring JIA patients suggests that once the inflammatory arthritic patients are judged to be in remission, most of them are monitored at 6–12 month intervals (115). This study also revealed that the assessment of remission relies more on the symptoms and plain radiography rather than MRI when following these patients over time, while, as already mentioned, the EULAR guidelines for JIA management claim MRI for both the diagnosis and follow-up monitoring (80). This supports the potential need for ongoing discussions between the Rheumatologist and Maxillofacial surgeons to determine the best imaging modality for individual patients (2, 115).

US may be possibly employed for monitoring scope through treatment course, even if, of note, randomized clinical trials of conventional and biologic disease-modifying anti-rheumatic drugs generally do not include TMJ as an outcome (16).

Conceivably, an association of clinical parameters and US details can be proposed as an integrative model.

A recent study by Johnston et al. (116) explored the link between TMJ inflammation as measured by US and patient disability as assessed by the Steigerwald Maher TMD Disability Index (SMTDI). This is the first study in which capsular width was integrated into a functional disability questionnaire.

## Conclusion

MRI is currently regarded as the gold standard imaging technique for the evaluation of TMJ pathology, as it can accurately identify both the active and chronic arthritic sequelae (65). This opinion is based on reliable parameters in terms of sensitivity and specificity from numerous studies and systematic reviews of the literature (21, 25, 28, 64); only the EULAR guidelines for JIA management recommend MRI for the diagnosis and follow-up evaluation of TMJ (80).

Regardless, US can be suggested as a useful examination tool in the assessment of TMD due to several advantages over MRI: low cost, large availability, and real-time quick assessment, the last two being favorable features, especially for claustrophobic patients and the pediatric population. Furthermore, US allows a dynamic and direct investigation of the surrounding structures (muscles, tendons, and ligaments), which is essential for an exhaustive understanding of the pathophysiological aspects of TMD and to obtain a first diagnostic approach to address a patient to more advanced imaging such as MRI after a positive screening when US is suspicious for TMD diagnosis (45).

However, as previously stated, given the potential for active and erosive TMJ arthritis in asymptomatic or minimally symptomatic patients, we should not disregard the value of suspicious US features (when the examination is not frankly positive for pathology but presents some unclarified signs). This situation can of course lead to unnecessary examination if MRI images do not confirm any pathological change, but reversely may earlier address the patient to appropriate management and early diagnosis of the pathological condition.

Noteworthy, the US survey is repeatable within a short time without any risk, allowing frequent monitoring of the pathology during the course of therapy, which is of particular relevance especially in children, avoiding the accumulation of TMJ damage.

**Figure 4:**
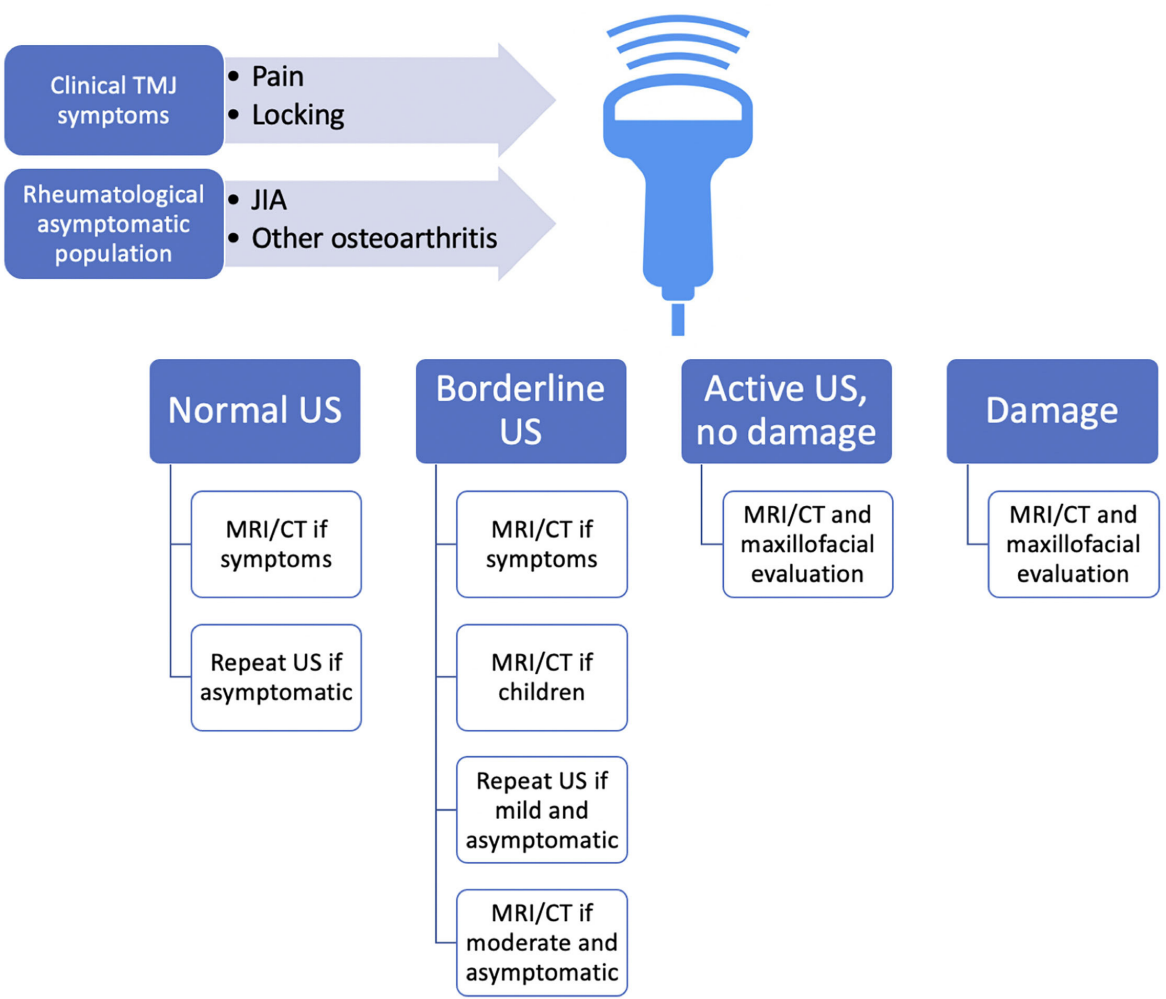
Overview of possible proposed employment of US in TMJ pathology screening as a first-level examination, guiding choice on the follow-up methods. As no validated protocol suggests any early imaging methodic screening in the rheumatological population at-risk for TMD, and also TMD may present rather asymptomatic during the early stages of the disease, we propose US as an “entry-level” method, which is rapidly accessible and of relatively low cost. US could approach all the patients with rheumatic conditions and hopefully also RA-at-risk patients, even if asymptomatic, and of course those with TMJ symptoms. Because US was found to be specific, but not particularly sensitive, we advocate MRI execution even for borderline suspicious findings at US, as baseline MRI could improve anatomic US accuracy during the follow-up. CT, computed tomography; JIA, juvenile idiopathic arthritis; MRI, magnetic resonance imaging; RA, rheumatoid arthritis; TMJ, temporomandibular joint; US, ultrasonography.

We have proposed a possible algorithm for US employment in TMJ pathology (Figure 4), which has no claim other than laying the groundwork for further reflection and development of studies that may hopefully clarify the importance of a preliminary analysis of TMJ through a non-invasive methodic such as US.

## Author Contributions

BM: conceptualization and writing the original draft preparation. BM, GC, SM, MGa, and MGo: methodology, investigation, data curation, writing, reviewing, and editing. MGa and MGo: supervision. All the authors have read and agreed to the published version of the manuscript.

## Funding

The APC was funded by the University of Ferrara.

## Conflict of Interest

The authors declare that the research was conducted in the absence of any commercial or financial relationships that could be construed as a potential conflict of interest.

## Publisher's Note

All claims expressed in this article are solely those of the authors and do not necessarily represent those of their affiliated organizations, or those of the publisher, the editors and the reviewers. Any product that may be evaluated in this article, or claim that may be made by its manufacturer, is not guaranteed or endorsed by the publisher.
